# A Homolog of Subtilisin-Like Proprotein Convertase 7 Is Essential to Anterior Neural Development in *Xenopus*

**DOI:** 10.1371/journal.pone.0039380

**Published:** 2012-06-28

**Authors:** Sema Senturker, John Terrig Thomas, Jennifer Mateshaytis, Malcolm Moos

**Affiliations:** Division of Cellular and Gene Therapies, Center for Biologics, Evaluation and Research, United States Food and Drug Administration, Bethesda, Maryland, United States of America; National Cancer Institute, United States of America

## Abstract

**Background:**

Subtilisin-like Proprotein Convertase 7 (SPC7) is a member of the subtilisin/kexin family of pro-protein convertases. It cleaves many pro-proteins to release their active proteins, including members of the bone morphogenetic protein (BMP) family of signaling molecules. Other SPCs are known to be required during embryonic development but corresponding data regarding SPC7 have not been reported previously.

**Methodology/Principal Findings:**

We demonstrated that *Xenopus* SPC7 (SPC7) was expressed predominantly in the developing brain and eye, throughout the neural plate initially, then more specifically in the lens and retina primordia as development progressed. Since no prior functional information has been reported for SPC7, we used gain- and loss-of-function experiments to investigate the possibility that it may also convey patterning or tissue specification information similarly to Furin, SPC4, and SPC6. Overexpression of SPC7 was without effect. In contrast, injection of SPC7 antisense morpholino oligonucleotides (MO) into a single blastomere at the 2- or 4-cell stage produced marked disruption of head structures; anophthalmia was salient. Bilateral injections suppressed head and eye formation completely. In parallel with suppression of eye and brain development by SPC7 knockdown, expression of early anterior neural markers (Sox2, Otx2, Rx2, and Pax6) and late eye-specific markers (β-Crystallin and Opsin), and of BMP target genes such as Tbx2 and Tbx3, was reduced or eliminated. Taken together, these findings suggest a critical role for SPC7–perhaps, at least in part, due to activation of one or more BMPs–in early patterning of the anterior neural plate and its derivatives.

**Conclusion/Significance:**

SPC7 is required for normal development of the eye and brain, possibly through processing BMPs, though other potential substrates cannot be excluded.

## Introduction

The role of Subtilisin-like Proprotein Convertases (SPCs) is well established in developmental processes, primarily by loss-of-function studies in various systems [1]. It is thought that their primary function in this context is to cleave growth factors belonging to the TGF-ß superfamily, at the recognition sequence RXXR, to their mature, biologically active forms. Previously, we evaluated the expression patterns of known *Xenopus* SPCs to determine which of these enzymes might play a role in patterning of the developing joint or body axis [2], [3]. We found that the mRNA expression pattern of *Xenopus* SPC7 (also called PC7) was inconsistent with a role in either of these processes. Instead, its localization during early development suggested the possibility that it might participate in patterning of specific anterior structures.

Here we report that loss of function of SPC7 in *Xenopus* leads to diminished anterior structures, including the mid- and forebrain, and anophthalmia (in which both lens and optic cup formation are suppressed). To assess how SPC7 fits into the developmental pathway controlling the formation of these structures, we examined the expression of several genes implicated in the early and late development of the brain and the eye. Since BMPs are among the best-studied SPC substrates and are known to participate in eye development, we also examined expression of BMP response genes and found it to be diminished. These results are consistent with a role for SPC7 upstream of the BMPs thought to be involved in early stages of brain and eye development.

## Results

### SPC7 is the Presumed Ortholog of Mammalian SPC7

**Figure 1:**
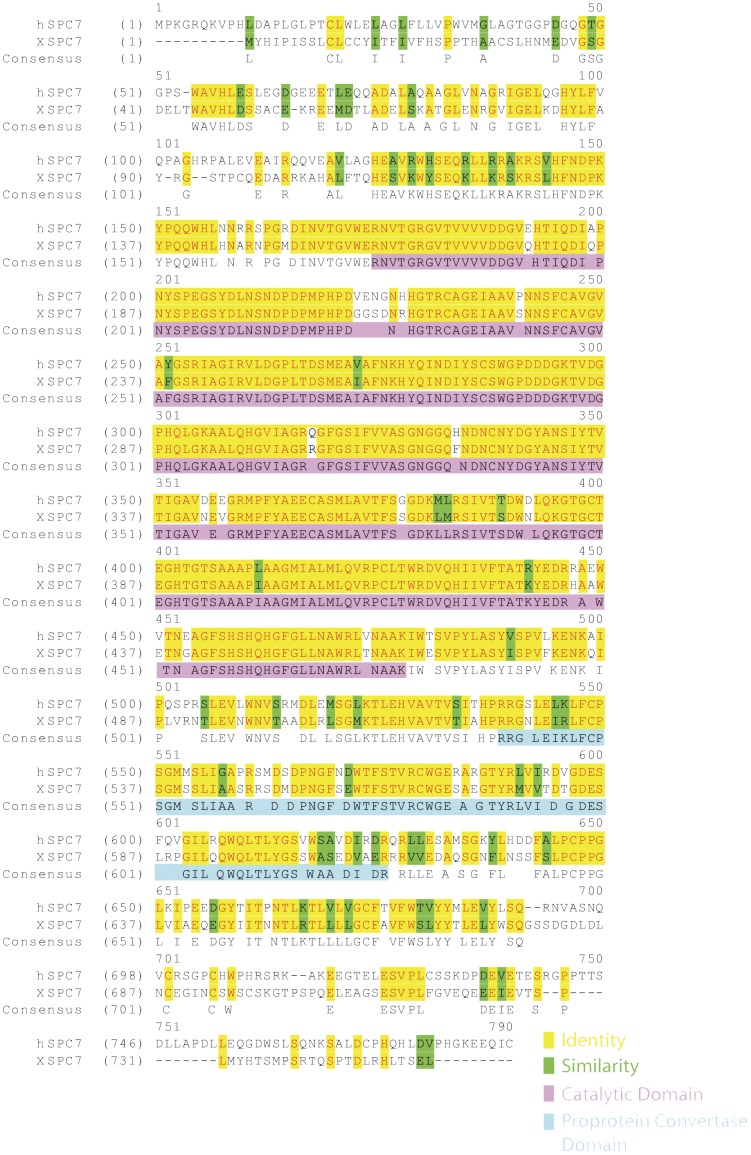
Sequence of SPC7. The amino acid sequence is aligned with that of human SPC7. Identities are highlighted in yellow, similarities in green, the catalytic domain in magenta, and the proprotein convertase domain in cyan.

The cDNA used in this study contained an open reading frame of 2,262 bp. The amino acid sequence is presented and compared with that of human SPC7 in Figure 1. The overall amino acid identity was 65% (91% in the catalytic domain, 68% in the proprotein convertase domain); all other SPCs were substantially less similar in primary sequence.

### SPC7 Shows Both Maternal and Zygotic Expression in Xenopus

**Figure 2:**
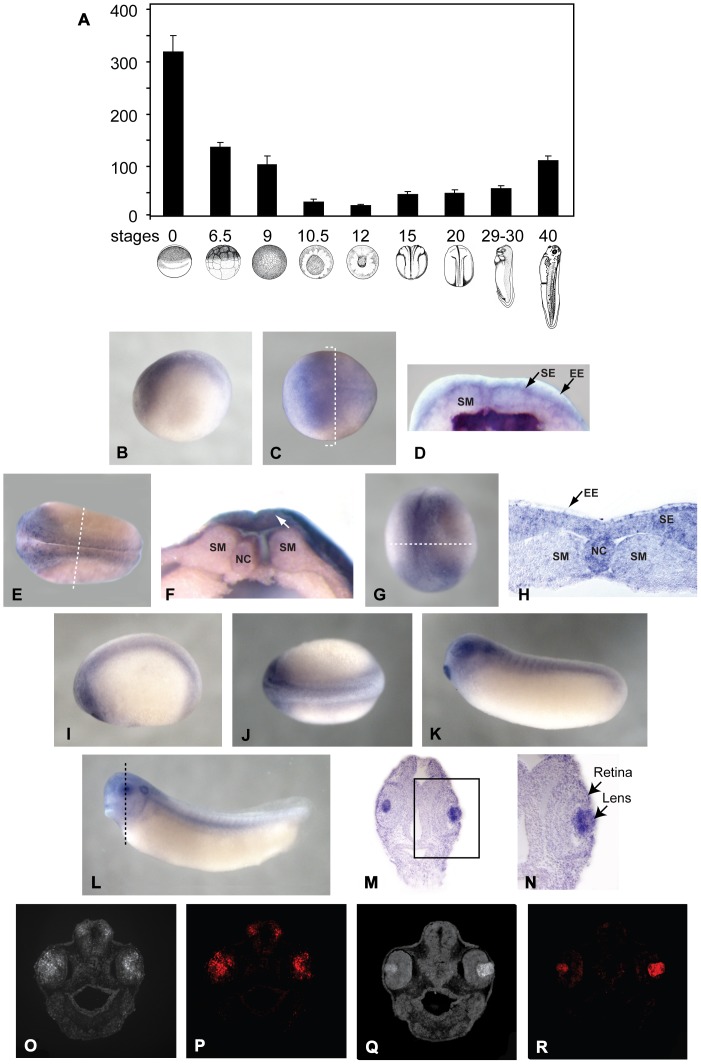
Expression of SPC7 mRNA in Xenopus embryos at different developmental stages. (A) Real-time PCR. (B-N) Whole mount hybridization *in situ* (in panels B, C, E, and I-L, anterior is to the left. Dotted lines in C, E, G, and L indicate the approximate plane for images in D, F, H, and M, respectively). (B-C) Lateral (B) and dorsal (C) views of a stage 13 embryo showing staining in the early neural plate. (D) Hemisected stage 13 embryo displaying detectable expression of SPC7 in the sensorial layer of the neuroectoderm, but not in the underlying somitogenic mesoderm. (E) Dorsal view of stage 16 embryo showing SPC7 staining in the neural folds and presumptive eye field. (F) hemisected midneurula (stage 16) showing staining in the sensorial ectoderm (arrow). (G) Antero-dorsal view of a late neural fold (stage 18) embryo showing staining in the anterior part of neural plate and anterolateral edges. (H) Plastic section through over-stained embryo showing staining in neuroectoderm. Lateral (I) and dorsal (J) views of a stage 21–22 embryo showing prominent staining in the eye, cement gland, brain, and neural tube. (K) Lateral view of stage 26 embryo showing SPC7 expression in the retina, lens, cement gland, otic vesicle, and throughout the head and somites. (L) At stage 32, staining in the lens became more prominent, primarily because expression in the retina decreased substantially. (M) Plastic section of over-stained stage 35 embryo showing specific SPC7 localization in the lens. (N) Enlarged view of boxed area shown in M. (O-R) Stage 30–32 (O, P) and stage 35–37 (Q, R) paraffin sections analyzed for SPC7. (O) Total fluorescence. (P) Same section as in O showing specific SPC7 staining in retina and lens (Fast Red). (Q) Total fluorescence. (R) Same section as in Q showing specific SPC7 fluorescent staining localized to the lens. (NC = Notochord; EE = Epithelial Layer of Neuroectoderm; SE = Sensorial Layer of Neuroectoderm; SM = Somitogenic Mesoderm).

Real-time PCR analysis revealed that SPC7 was expressed from stage 0 to 40 (the latest stage analyzed, Figure 2A). The highest SPC7 mRNA level was detected in eggs; expression then decreased until midgastrulation and increased thereafter.

Whole-mount hybridization *in situ* demonstrated SPC7 expression in the early neural plate at stage 13 (Figure 2B-C). The SPC7 signal for early stage embryos was too faint for analysis of histological sections; expression in various germ layers was evaluated in hemisected embryos to obviate this. The stage 13 hemisected embryo shown in Figure 2D displayed detectable expression of SPC7 in the ectoderm, while no staining was evident in the underlying mesoderm. By the mid to late neural fold stage (Figure 2E-H), expression was apparent in the neural and head folds; punctate nuclear staining in 2E and H, consistent with transcriptional bursts, was prominent in scattered cells. Sections cut through stage 18 embryos embedded in JB4 resin displayed similar staining patterns to the stage 13 hemisected embryos we evaluated; staining was evident in the sensorial layer of the neuroectoderm, but not in the underlying somitogenic mesoderm (Figure 2H). By stage 22, staining was observed in the eye, cement gland, brain, and neural tube (Figure 2I-J). At stage 26, increased staining intensity was evident in the retina, lens, cement gland, otic vesicle, and somites (Figure 2K). By stage 32, SPC7 staining remained in these structures (Figure 2L). At this later stage the staining became more prominent in the lens, primarily due to increased staining contrast via decreased retinal expression (Figure 2L). To further evaluate these findings over-stained embryos from the whole mount in situ experiment were embedded in JB-4 resin and sectioned through the head, revealing that at later stages (35–37) SPC7 staining had become specific to the lens of the eye (Figure 2M-N). These data were confirmed by hybridization to paraffin sections (Figure 2O-R).

### SPC7 Loss of Function Results in Diminished Anterior Structures and Anophthalmia

Injection of 300 pg SPC7 mRNA into a single blastomere at either the two or four cell stage produced no detectable abnormalities in phenotype (data not shown), consistent with the expectation that constitutive gene expression levels are sufficient to promote maximal cleavage of the enzyme’s substrate(s). We used antisense morpholino oligonucleotides (antisense-MOs; reviewed in [4], [5]) to evaluate loss of SPC7 function.

**Figure 3:**
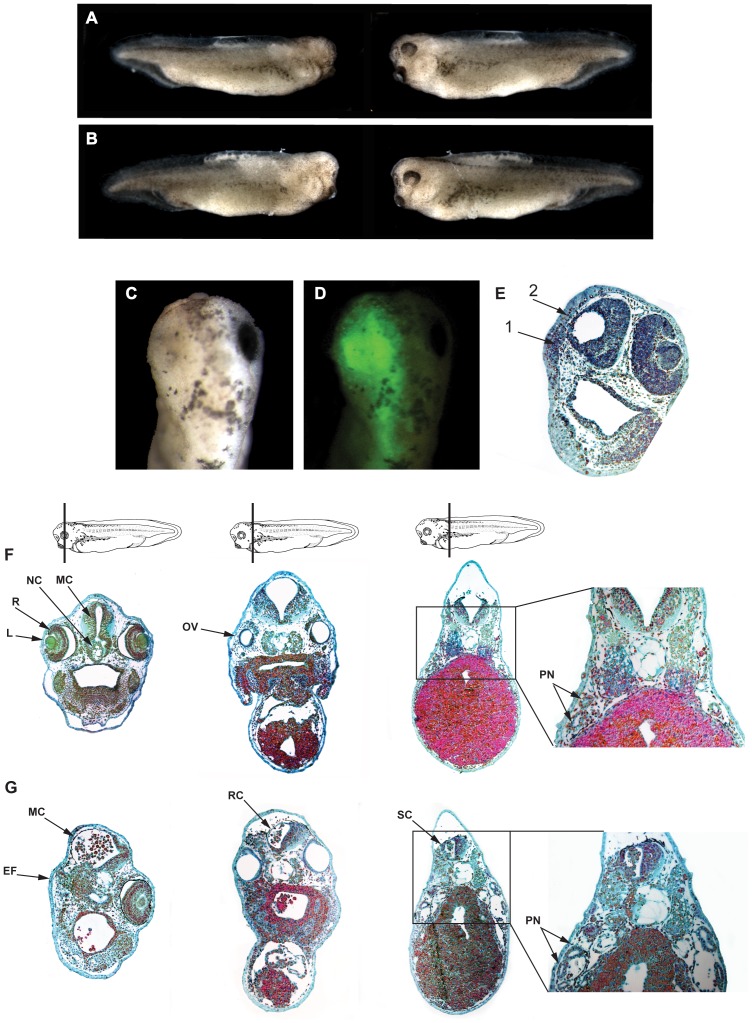
SPC7 antisense morpholino oligonucleotide causes dose-dependent disruption of eye and brain development. (A, B) Embryos injected unilaterally with antisense SPC7-MO at the two cell (A) or four cell stage (B). Injected side is shown on the left. Both the two cell and four cell stage loss-of-function SPC7 experiments resulted in the same phenotype: lack of eye, lack of branchial arches, and diminished head structures on the MO-injected side. The morphology of the rest of the embryo appeared normal. (C) Dorsal view of tadpole-stage embryo injected at the four cell stage with 90 ng MO; the eye appears to be completely absent on the injected side. (D) Same embryo as in C showing MO location on the left. (E) Frontal section of embryo in C and D showing total loss of eye and mesencephalon on the SPC7-MO injected side. The uninjected side was unaffected. Arrows 1 and 2 show presumptive eye field and mesencephalon, respectively, on the MO injected side. In each experiment, embryos injected with control MO developed normally (not shown). (F, G) Histological analysis of control-MO injected (F) and SPC7-MO unilaterally-injected (G, injected side to the left) stage 35 embryos. The planes of section presented are shown diagrammatically at the top. As noted previously, the eye and brain are severely dysmorphic on the injected side; the otic vesicle and pronephros appear normal. (L =  Lens; R = Retina; NC = Notochord; MC = Mesencephalon; RC = Rhombancephalon; SC =  Spinal Cord; OV = Otic Vesicle; PN = Pronephros; EF = Eye Field).

We examined the effects of unilateral injections of SPC7 antisense-MO at the two cell stage and also at the four cell stage; results were identical for both (Figure 3A, B). The effect of the SPC7 antisense-MO was dose-dependent. At the 30 ng dose (Figure S1), we observed a small eye phenotype in 68% - 75% of the injected embryos (5 separate experiments; a total 143 of the 196 viable injected–i.e., fluorescein positive–embryos). At the 30 ng dose, lens tissue was totally absent in all embryos examined, but small clusters of cells without discernable retinal layers were present (Figure S1C). For the 90 ng dose (8 separate experiments), a totally anophthalmic phenotype was observed in 83%–90%, or 239 of 267 total viable injected embryos. At this dose the effects were more pronounced; eyes were essentially completely absent, though small layers of cells consistent with optic cup rudiments could be discerned in some embryos. The mesencephalon was also absent on the injected side (Figure 3A-E). Histological evaluation of embryos injected with 90 ng control-MO or SPC7 antisense MO is presented in Figure 3F and Figure 3G, respectively. Sections taken through the entire embryo were examined in each case. As noted previously, the eye and brain were hypo- or dysmorphic in injected embryos, but the otic vesicle and pronephros appeared normal, as did all more posterior structures. Since no nonspecific toxicity was observed at 90 ng, all subsequent experiments–including all rescue experiments done to exclude possible off-target effects–were conducted with 90 ng MO.

Bilateral injection of SPC7-MO (both dorsal blastomeres at the two or four cell stages) completely suppressed formation of externally visible head structures in all embryos examined (3 separate experiments; 91 viable injected embryos); in histological sections, both eyes and the midbrain were absent (data not shown). Bilateral injection of control morpholino had no observable effect.

All of the experiments were repeated with a second SPC7 antisense morpholino oligonucleotide and the results were identical (2 separate experiments; 70 of 86 viable embryos evaluated displayed the eye phenotype). The control MO had no effect: of 159 fluorescein positive embryos evaluated, none showed detectable abnormalities.

### Loss of Function Effect of SPC7 Antisense Morpholino Oligonucleotide is Specific

**Figure 4:**
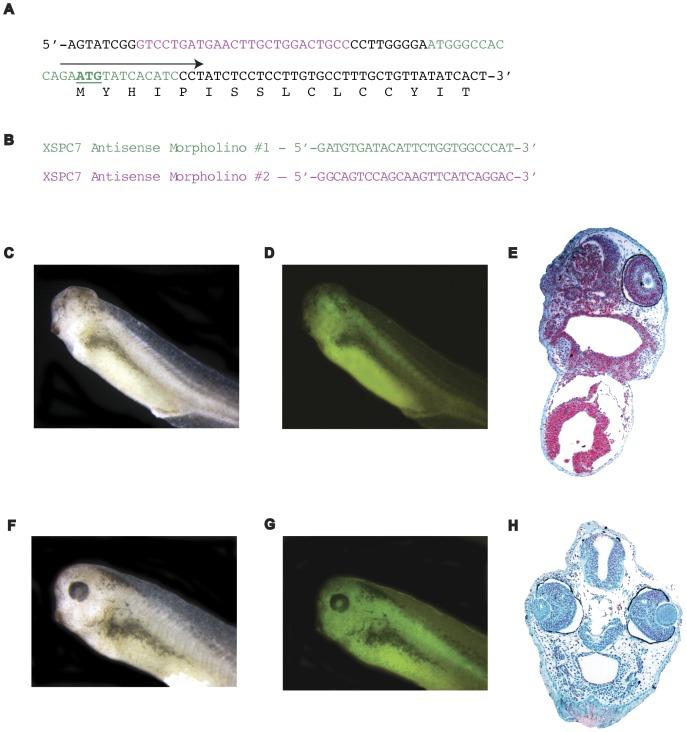
5′ sequence of SPC7. (A) 5′ sequence of SPC7 and antisense morpholino oligonucleotides. Sequences corresponding to SPC7 MOs 1 and 2 are shown in green and magenta, respectively. Start codon is underlined. Arrow denotes 5′ primer used to construct synthetic mRNA used in rescue experiments. (B) Sequences of antisense morpholino oligonucleotides. (C, D) Bright and dark field images of stage 37 SPC7 antisense morpholino-only control embryo showing anophthalmic SPC7-MO phenotype on injected side. (E) Histological section through SPC7-MO embryo; note lack of distinguishable eye structures on the injected side and severe mesencephalic hypoplasia. (F, G) Bright and dark field images of stage 37 embryo coinjected with SPC7 antisense morpholino oligonucleotides and synthetic rescue mRNA, showing normal morphology on the injected side. For all rescue experiments, we used the 90 ng dose. Complete rescue as shown in (F-H) was observed in all embryos in each of three separate experiments (106 total embryos were scored); identical results were obtained with either SPC7-MO. (H) Histological section through coinjected embryo; both eyes appear normal.

To confirm the specificity of SPC7 antisense-MO further, we created a truncated *Xenopus* SPC7 mRNA that could not hybridize to either of the antisense-MOs we tested (Figure 4A, B). 300 pg of this mRNA were able to rescue the eyeless phenotype completely in all embryos examined when coinjected with either of the anti-sense morpholinos (Figure 4F-H). As indicated previously, only the 90 ng dose was used in the rescue experiments (3 separate experiments; a total of 106 healthy injected embryos were scored; eye abnormalities could not be detected in any embryo injected with the truncated SPC7 mRNA and antisense-MO). Results were identical with both morpholino oligonucleotides tested. Controls injected with SPC7-MO alone were included routinely to verify that the morpholino was active (Fig. 4C-E).

### Loss of Function of SPC7 Does Not Affect Mesodermal and Posterior Neuroectodermal Marker Expression Patterns

**Figure 5:**
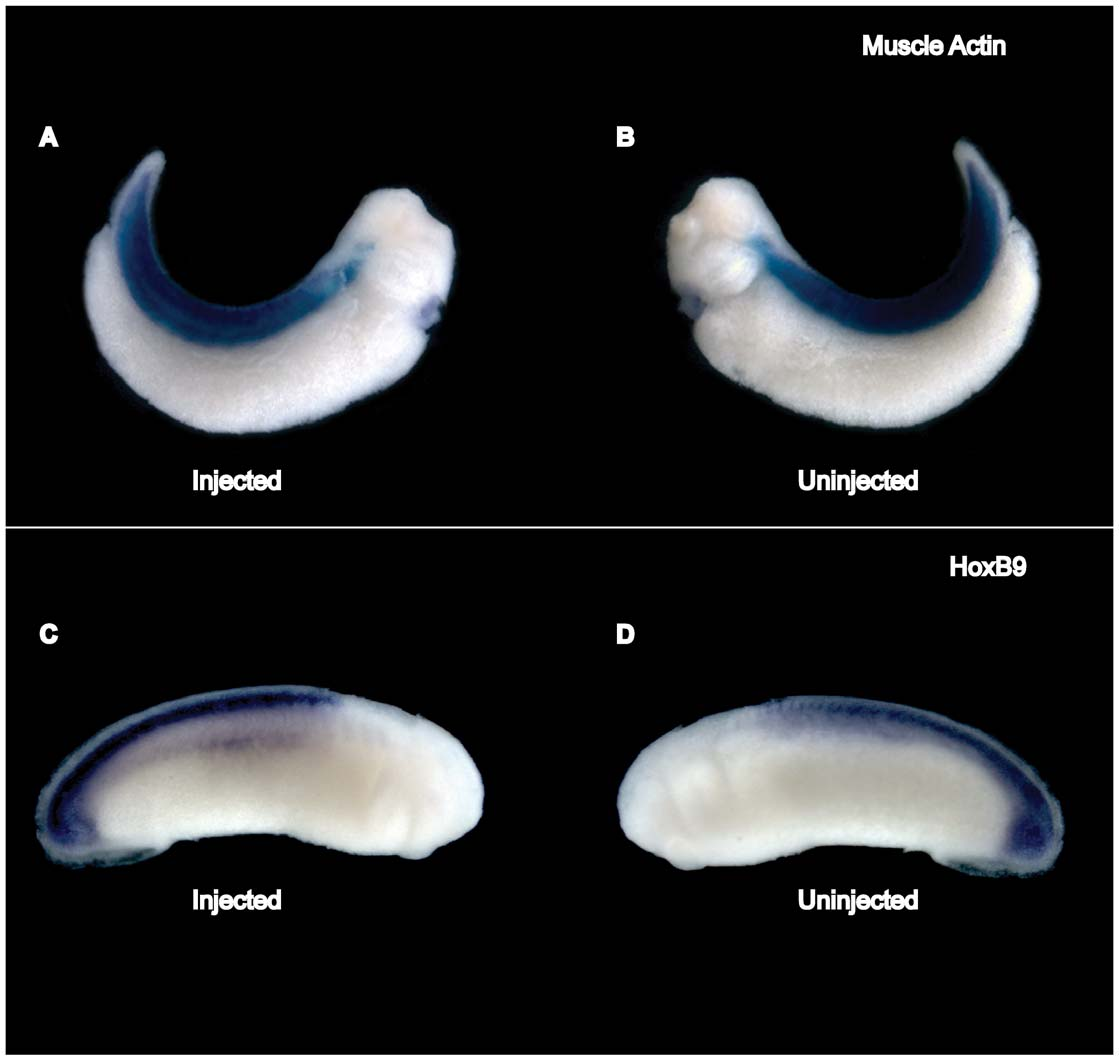
Loss of SPC7 function does not affect mesodermal and posterior neuroectodermal marker expression patterns. Whole mount hybridization *in situ* of embryos injected unilaterally with 90 ng SPC7 morpholino. Injected sides are shown to the left. (A, B) Lateral views of stage 35 embryo showing that muscle actin (dorsal mesoderm) staining was equally visible in both the injected and uninjected sides of the embryo. (C, D) Lateral views showing that HoxB9 (posterior neuroectoderm) expression was equally apparent in the posterior dorsal region of the embryo in both the injected and uninjected sides.

We evaluated the regional specificity of SPC7 action by examining the expression of posterior neuroectodermal (Hox B9) and mesodermal (Muscle Actin) markers in SPC7- MO unilaterally injected embryos. Both the injected and uninjected sides of the embryo showed normal expression patterns for these markers (Fig. 5A-D; embryos shown here were injected at the four cell stage).

### SPC7 Loss of Function Suppresses Markers for Anterior Patterning of Neural Tissues and Early Eye Development

Many transcription factors have been implicated in the anterior patterning of neural tissues including the eye [6], [7], [8], [9]. These include: Sox2 (sex determining region Y-box 2), which is expressed in the anterior neural plate and placodal tissues and is essential for crystallin promoter activation [10], [11]; Rx2a (retinal homeobox2a), an early eye marker essential for specification, survival, and proliferation of retinal cells in eye development [7], [8]; Otx2 (orthodenticle homeobox 2), one of the earliest genes to originate from the anterior neural plate [6], [10]; and Pax6 (paired box gene 6) [12], the vertebrate homolog of Drosophila *eyeless* [13]. These have all proven to be useful as early markers for brain and eye specification. Opsin, a major protein in the photoreceptor cells, is a useful marker for terminal differentiation of the retina; it first appears in *Xenopus* at stage 29 and is expressed thereafter [14]. β-Crystallin is the major structural protein of the lens, and so is a well- established marker for the differentiating cells within the posterior lens vesicle, which first becomes detectable at stages 26–27, and for the mature lens [15].

**Figure 6:**
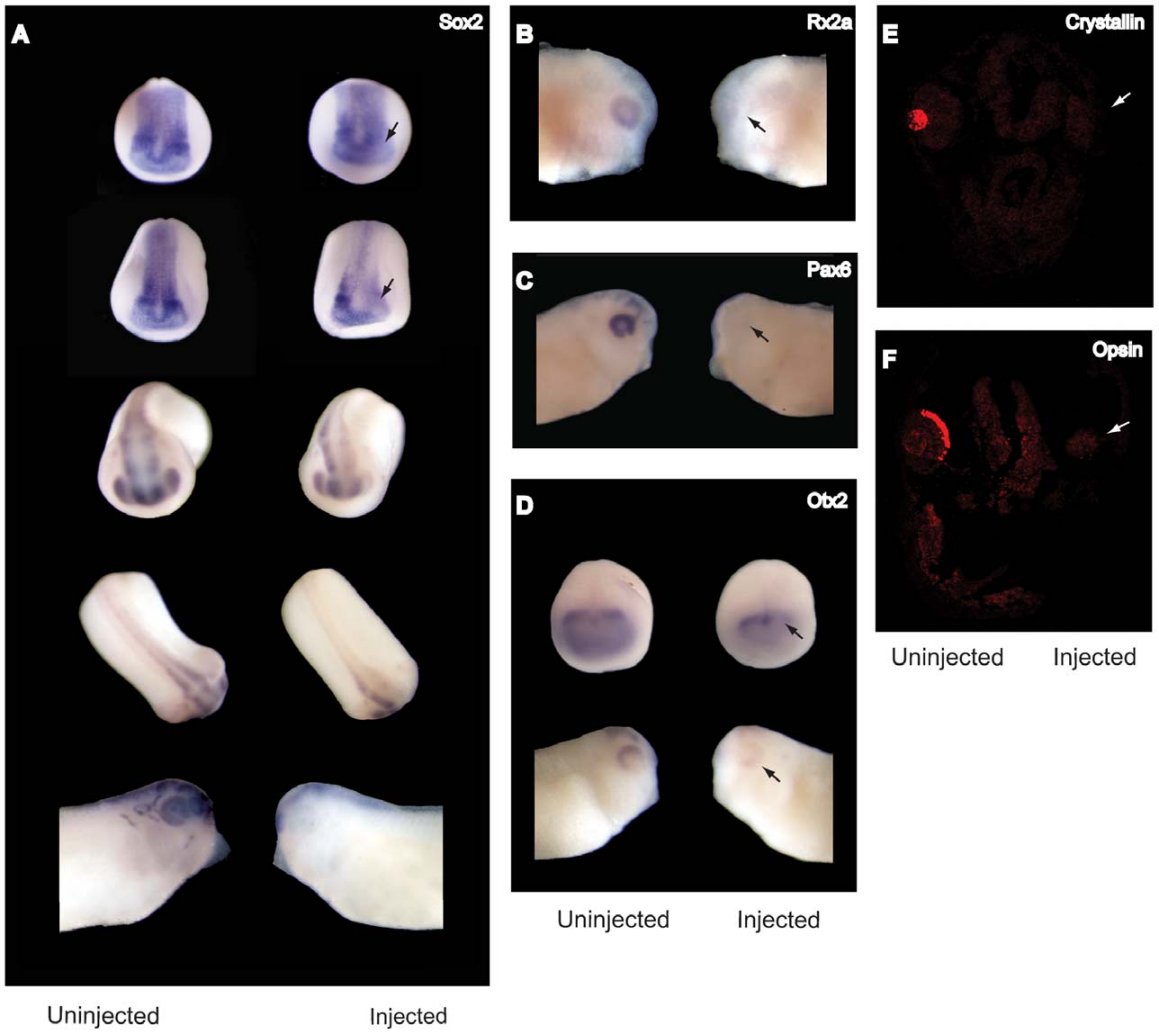
Loss of function of SPC7 diminishes expression of neural and eye markers. (A-D) Whole mount hybridization in situ of embryos injected unilaterally with 90 ng SPC7 morpholino. Injected sides are shown to the right. (A) Frontal, dorsal, and lateral views showing Sox2 expression in control (left) and SPC7-MO unilaterally injected (right) embryos. Early- (top row) and mid-neurula (second row) embryos showing diminished Sox2 staining on the SPC7 morpholino injected side, most obviously in the eye anlage but apparent throughout the head folds (arrows). The loss of expression became more obvious at later stages. Row Three: antero-frontal view of stage 22 embryos. Sox2 staining was undetectable in retina, lens, and neural tube, and was reduced in the midbrain on the injected side. Row 4: dorsal view of the same embryos showing that Sox2 staining was absent in the neural tube on the injected side. Bottom row: Stage 35 embryo showed complete loss of Sox2 staining in the eye on the injected side. (B) Lateral views showing Rx2a expression was not detectable on the injected side (arrow). (C) Lateral views showing Pax6 expression pattern in control (left) and injected (right) stage 26–27 embryos. Pax 6 staining was absent in the eye field on the injected side (arrow). (D) Otx2 expression in uninjected control (left) and SPC7-MO unilaterally injected (right) embryos. Top row: antero-dorsal view of stage 14 embryos showing significantly diminished Otx2 expression on the injected side (arrow). Bottom row: Lateral views of control (left) and SPC7 MO-injected (right) sides of the same embryo showing barely detectable Otx2 expression on the injected side (arrow). (E, F) Frontal sections through the head of stage 35 embryos injected unilaterally (injected side to the right) with 30 ng SPC7-MO showing expression of β-Crystallin (E) and Opsin (F) expression on the non-injected side only.

To evaluate molecular correlates of the phenotype, we examined these markers in embryos injected unilaterally with SPC7-MO by hybridization in situ. Sox2 expression in neurula stage embryos was substantially decreased on the injected side, compared with the uninjected side or with the control-MO injected embryos (Figure 6A). In later stage embryos, Sox2 expression in the retina was nearly absent on the injected side; in the midbrain and neural tube, staining was markedly reduced compared with the uninjected side or with control-MO injected embryos (Figure 6A). Similarly to Sox2, Otx2 expression in the MO-injected side was markedly reduced in neurulas, and undetectable in the eye and midbrain of somite and tadpole stage embryos; the contralateral uninjected side and control-MO injected embryos showed the normal expression pattern (Figure 6D). Rx2a was undetectable in the retina on the injected side, while the uninjected side showed normal expression (Figure 6B). Results were similar for Pax6; SPC7-MO significantly suppressed observable staining in the eye and caused reduced expression in the midbrain (Figure 6C).

Consistent with the near total lack of eye tissue observed at the 90 ng MO dose, β- Crystallin and Opsin transcripts could not be detected on the injected side of stage 33–35 embryos by hybridization in situ in frontal sections through the eye field (not shown). Since some presumptive optic vesicle tissue remained at the 30 ng dose (Figure S1C), we also evaluated expression of both markers in these embryos. Here also, expression of these terminal differentiation markers was undetectable on the injected side, while the expression pattern on the uninjected side and in control-MO injected embryos was normal (Figure 6E, F).

### SPC7 Loss-of-Function Suppresses BMP Response Genes

**Figure 7:**
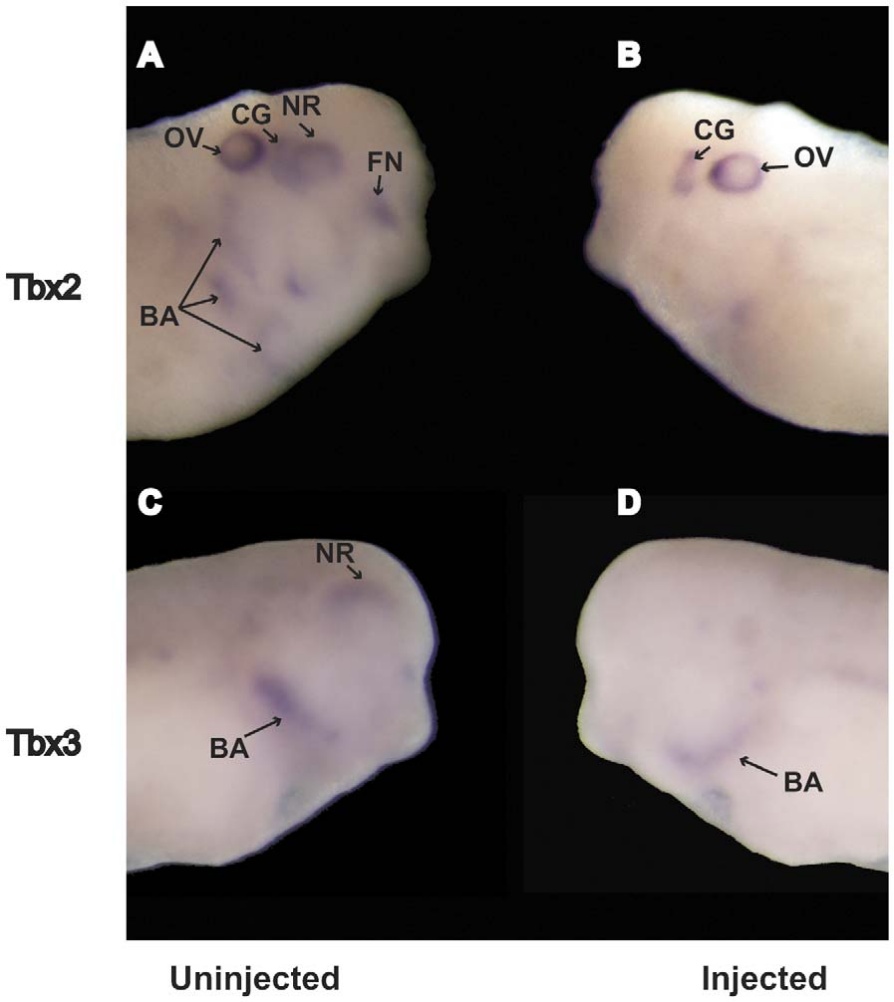
BMP target genes Tbx2 and Tbx3. Whole mount hybridization in situ of BMP target genes Tbx2 and Tbx3 in stage 33/34 embryos injected unilaterally with 90 ng SPC7 morpholino. Injected sides are shown to the right. (A, B) Lateral view of embryos showing Tbx2 expression was lost in the eye on the MO-injected side only (NR, neural retina; CG, cranial ganglion; OV, otic vesicle; BA, branchial arches; FN, frontonasal process). (C, D) Lateral view of stage 33/34 embryos showing a similar result for Tbx3.

One well-appreciated function of the SPCs is to process BMPs [16], which are thought to act early in the sequence of events leading to specification of anterior neural structures including the eye [9]. We therefore evaluated BMP signaling with hybridization in situ for the BMP response genes Tbx2 and Tbx3 [17], [18] which are closely related T-box proteins that have been implicated in development of a number of different tissues including the eye [19]. SPC7-MO injection resulted in total loss of Tbx2 expression in the eye, frontonasal region, and branchial arches; expression in the otic vesicle and cranial ganglia was unaffected (Figure 7A, B). Tbx3 expression was diminished in the branchial arches on the MO-injected side, and was totally undetectable in the eye region (Figure 7C, D).

## Discussion

The importance of the Subtilisin-like Proprotein Convertases in controlling the activity of TGF-ß superfamily proteins is now well-established [16]. Recently, we presented evidence that anatomically restricted overlap of the expression domains of these proteases and growth factors provides an additional level of regulation. In these studies, we evaluated the potential of known SPCs to participate in patterning of the limb [3] and body axis [2]. SPC7 was excluded on the basis of its temporal and spatial expression patterns; its expression in the limb cannot be detected by RT-PCR, and SPC7 is expressed in the blastula at a site remote from the dorsal vegetal endoderm [2], [3]. However, its discrete expression pattern suggested the possibility that this protease might participate in specification of anterior structures (Figure 2).

The loss of function data presented here indicate a requirement for field- specific processing by SPC7 during development of the eye and brain; unilateral injection of antisense morpholino oligonucleotides produced dose-dependent loss of eye and brain tissue. At the 90 ng dose, both lens and optic vesicle were absent in all embryos evaluated (Figure 3E, G), and the brain was severely hypomorphic; at 30 ng, the lens was absent but dysmorphic optic vesicle tissue was present in some embryos (Figure S1C). We consider non-target effects unlikely, since 1) injection of control MO was consistently ineffective; 2) similar results were obtained with two different non-overlapping antisense SPC7 oligonucleotides; and 3) mRNA rescue of embryos injected with the 90 ng dose of either MO oligonucleotide was complete (Figure 4A-E) in every single embryo examined.

The near total suppression of the terminal eye differentiation markers crystallin and opsin was consistent with the gross and histological findings. Moreover, reduced expression of early markers of brain and eye development (Sox-2 and Otx-2) by the SPC7 antisense oligonucleotides in neurula stage embryos (Figure 6) suggests that SPC7 is essential near the beginning of events leading to specification of the anterior neural plate. Suppression of these markers, as well as the BMP response genes Tbx2 and Tbx 3 (Figure 7), would be consistent with existing models [7], [9] that place BMP signaling proximal to these transcription factors in the specification of the anterior nervous system. Thus, the absence of eye and brain resulting from SPC7 knockdown that we observed likely resulted from a more general disruption of these early patterning events.

Approximately 3500 mammalian secretory proteins, including growth factors and their receptors, are subjected to limited proteolysis by one or more members of the SPC family [20]. The complexity of substrate processing, based on redundancy and specificity of SPCs, is variable depending on the tissue. The well established role of SPCs for BMP processing in many developmental processes and the widely-appreciated requirement for BMP signalling in eye development prompted us to examine these factors.

Our data cannot exclude the possibility that SPC7 may act on non-BMP substrates that play a role in eye and/or forebrain development. Nevertheless support for the involvement of BMPs in these processes is substantial. BMP4 and BMP7 are both known to be substrates of SPC7. They are required for eye patterning and are thought to act in early stages of this process. BMP7 gene targeting experiments in the mouse lead to a variable phenotype that ranges from mild microphthalmia to anopthalmia [21], [22]. In severely affected null mice, the lens placode fails to form and the placodal phase of Pax6 expression is lost [23]. These findings indicate that BMP7 plays an important role upstream of Pax6 during lens placode formation and specification. BMP4−/− null mutants exhibit defects in lens induction and lens-specific Sox2 expression is diminished [24]. Even though BMP7 and BMP4 have overlapping expression patterns in the eye and BMP7−/− and BMP4−/− mice display a similar lens phenotype, there are some distinct differences between these two mutants. In particular, Pax6 expression is lost in BMP7−/− mutants [23] but not in BMP4−/− mice [24]. These observations indicate that BMP4 and BMP7 may have non-redundant functions in eye development [9]. SPC7 loss-of-function significantly decreased eye-specific expression of Pax6 (Figure 6D). Therefore, the action of SPC7 cannot be explained on the basis of BMP4 activation alone; one possibility is that it also is required for processing of BMP7 in the anterior neural fold. Moreover, both BMP4 and BMP7 null mice form optic cup tissue [21], [24], whereas at high doses of SPC7-MO, these tissues are absent. Another BMP, Growth and Differentiation Factor 6 (GDF6) may also be a substrate for SPC7; GDF6 loss-of-function produces small eyes in *Xenopus* [25]. However, since the SPC7 loss of function phenotype we observed is much more severe than that reported for GDF6- depleted embryos, it is clear that this BMP cannot be its only substrate. Thus, it is unlikely that the apparent requirement for SPC7 can be explained by its action on any one of these growth factors alone, but rather on some combination of BMP4, BMP7, and possibly GDF6, as well as other yet undetermined substrates. As indicated previously, it is possible that non-BMP substrates for SPC7 (e.g., pro-EGF, [20]) may also be important in eye and/or forebrain development, but there is currently little direct evidence for this.

Thus, coexpression of SPC7 with BMPs required in anterior neural development may represent another situation in which one level of control over growth factor activity is exerted by virtue of anatomically restricted processing of growth factors to a specific developmental field. This is analogous to our earlier findings with CDMP-1/GDF-5 [3] and Vg1 [2]. There is a description of unpublished data indicating that SPC7 loss of function does not produce a phenotype in mice [26]. Our data make it clear that the situation is different in *Xenopus*, so the notion that SPC7 is not required during development cannot be generalized to other vertebrates. However, both BMPs and SPCs have substantial functional redundancy [16], and it is possible that such compensatory mechanisms may be more advanced in mammals than in amphibians. Resolution of this question may be an appropriate topic of further study.

## Methods

### Embryo Manipulations

Frogs and their embryos were maintained and manipulated using standard methods [27], [28]. All embryos were staged according to Nieuwkoop and Faber [29] and Keller [30]. Injection experiments were performed by standard procedures as described previously except embryos were maintained in 0.3x MMR supplemented with 3% Ficoll 400 [31] during and immediately following injection. Dorsal and ventral blastomeres were identified by size and pigment variations [29]. All injection experiments were carried out at both the two and four cell stage unless otherwise noted. For unilateral injections, mRNA and morpholino-antisense oligonucleotides were injected into a single dorsal blastomere. For bilateral injections, injections were into two blastomeres at the two or four cell stage. All loss-of-function experiments included control-MO injected embryos. Except as noted, all MO injections (including rescue experiments) were done at the 90 ng dose, and routinely included embryos injected with control oligonucleotide (or mRNA where appropriate).

Embryos were photographed with low angle oblique illumination and a Zeiss Stemi-6 dissecting microscope or with a Leica DCF420 camera.

### mRNA Quantitation (Real-Time PCR)

Total RNA was extracted using Trizol (Invitrogen) or the RNeasy kit (QIAGEN). Quantitative real-time PCR analyses were performed using the ABI 7900 sequence detection system. SPC7 and Histone specific primers were designed using Primer Express software (SPC7 forward primer 5′-CTG CGA GAA AAG GGA GGA GAT-3′, reverse primer 5′-GCC CTG TGG CTT TGG AAA-3′. Histone forward primer 5′-ACG CCG TCA CCT ACA CAG AAC-3′, reverse primer 5′-ACC ACA TCC ATG GCG GTA AC-3′). SPC7 and Histone mRNA samples were first reverse transcribed and then subjected to SYBR Green quantitative real-time PCR. Known quantities of 10-fold dilutions of total input cDNA were used to generate standard curves for each primer pair. Relative amounts of each embryonic stage were determined in the linear range according to their C_T_ value. For each primer set, melting curves were used to verify the correct PCR product. Histone H4 mRNA levels were used for normalization. All PCRs were performed in triplicate.

### Plasmids and Probes

The *Xenopus* SPC7 plasmid was as described previously [3]; Genbank accession number: EU879966). The following ESTs were purchased from the Open Biosystems: Sox2 (clone id 3398743), Rx2a (clone id 7981599), Otx2 (clone id 6632834), Pax6 (clone id 79800371), β-Crystallin (clone id 6879338), Opsin (clone id 7019321), Tbx2 (clone id 6865037), Tbx3 (clone id 7980218), muscle actin (clone id 4409317), and HoxB9 (clone id 7982206). The poly A tails were removed with HindIII for Opsin and Sox2 or with ApaI for Rx2a. The plasmids were then religated. For Otx2 and Pax6, the poly A tail was removed by digestion with SalI/BamHI or EcoRV/XhoI, respectively. The poly A tail of Tbx2 was removed by EcoR1 digestion; the insert was then subcloned into PCR4-TOPO (Invitrogen) and sequenced. For Tbx3, the poly A tail was removed by digestion with EcoRV/Not1 and the plasmid religated. Finally, the Poly A tails were removed with Kpn1 for muscle actin, or with PvuII and Not1 for HoxB9. The plasmids were then religated. The β-Crystallin EST did not contain convenient restriction sites; we therefore amplified the open reading frame by PCR using following primers: Forward, 5′-TAC CGG ACA TAT GCC CGG GAA-3′ and reverse, 5′-GCA TCA TAT TGT AAA GTA CAT CGG T-3′. The product was subcloned into PCR4-TOPO. Correct sequence and orientation were confirmed by bidirectional sequencing of all plasmids.

### Antisense Morpholino-oligonucleotides and Rescue Constructs

*Xenopus* SPC-7 antisense (we used both 5′- GATGTGATACATTCTGGTGGCCCA T-3′ and 5′-GGCAGTCCAGCAAGTTCATCAGGAC-3′ with similar results) and control (5′-TACCCGGTGGTCTTACATAGTGTAG-3′) morpholino oligonucleotides, tagged with fluorescein, were purchased from Gene Tools. For mRNA rescue experiments, we amplified the open reading frame of SPC7 with the primers: forward, 5′-GAA TGT ATC ACA TCC CTA TCT CCT -3′; reverse, 5′-TGC CAC ATT CAG TCA TAT CAG GGT-3′. The resulting cDNA contained the complete open reading frame but lacked 11 bp of 5′-UTR sequence present in the SPC7 morpholino oligonucleotides and so could not form a stable hybrid with either morpholino. This was subcloned into pCS2 and the sequence verified as above.

### Histology

Embryos were embedded in JB-4 resin according to manufacturer’s instructions, sectioned on a Leica RM2265 rotary microtome at 2 or 5 microns, and mounted on SuperFrost Plus slides (Fisher) to facilitate adherence during staining. To accentuate the eye and clearly differentiate neural retina and lens from other tissues, a method based on the Feulgen reaction followed by counterstaining with Light Green and Orange G [32] was modified for use in plastic sections as follows. Sections were rinsed in 1 N HCl, hydrolyzed for ten minutes in 1 N HCl at 60°C, and rinsed at RT in 1 N HCL followed by water. The sections were then stained in Schiff reagent for 24–48 hours or in acidified alcoholic basic fuchsin [33] for 30 minutes. Stained sections were drained, rinsed 3 times in 0.5% potassium metabisulfite in 5% HCL, and then rinsed with tap water. They were then fixed on a hot plate at 80°C for at least 10 minutes. Sections were counterstained with Light Green (0.2% in 95% ethanol) for 20 seconds followed by a water rinse, and then with Orange G (0.2% in 0.2% phosphotungstic acid ) for 40 seconds. The staining times were adjusted as needed to achieve optimum color differentiation. Finally, slides were rinsed with water and air dried.

### Hybridization in Situ

cRNA probes were produced using MEGAscript T3, T7, or SP6 in vitro transcription kits (Ambion), incorporating digoxigenin. For whole mount hybridization in situ on *Xenopus* embryos, procedures outlined by Harland were followed [34], with modifications as described [31]. For colorimetric detection, signals were developed using alkaline-phosphatase conjugated antibodies to digoxigenin and BM-Purple (Roche). Overstained embryos were embedded in JB-4 resin (Polysciences, Warrington, PA) after abbreviated infiltration (3×10 min) and sectioned at 20 microns with a Leica RM2265 rotary microtome.

Paraffin sections (7–10 µm) were prepared for hybridization in situ using a standard protocol [35]. Prior to hybridization, sections were incubated for 30 min at 90°C in 10 mM citrate buffer, pH 6.0 to enhance antigenicity [36]. Hybridization was performed at 60°C overnight in the presence of 1 µg/ml of probe. For fluorescent detection, probes were labeled with digoxigenin and a sheep alkaline- phosphatase conjugated anti-digoxigenin antibody (Roche) was used in combination with Fast™ Red (Sigma) fluorogenic substrate. Confocal images (crystallin and opsin) were obtained using a confocal Zeiss Imager Z1microscope with a krypton-argon and blue diode laser. SPC7 detection was done with a Nikon Eclipse 80i using MetaMorph image analysis software.

## Supporting Information

Figure S1 **SPC7 antisense morpholino oligonucleotide causes dose-dependent disruption of eye and brain development –30 ng dose.** (A) Dorsal view of the head of a stage 35 embryo injected unilaterally with 30 ng MO. The MO injected side, to the left, failed to develop a normal eye. (B) Localization of fluorescein tagged MO in same embryo. (C) Frontal section of embryo shown in (A) and (B) showing a disorganized neural retina and no lens (arrow). The right, uninjected side was unaffected.
(TIF)
