# Supplementary figures and images for: A Homolog of Subtilisin-Like Proprotein Convertase 7 Is Essential to Anterior Neural Development in Xenopus
Source: PLoS One. 2012 Jun 28;7(6):e39380. doi: 10.1371/journal.pone.0039380 (PMC3386266; doi:10.1371/journal.pone.0039380)

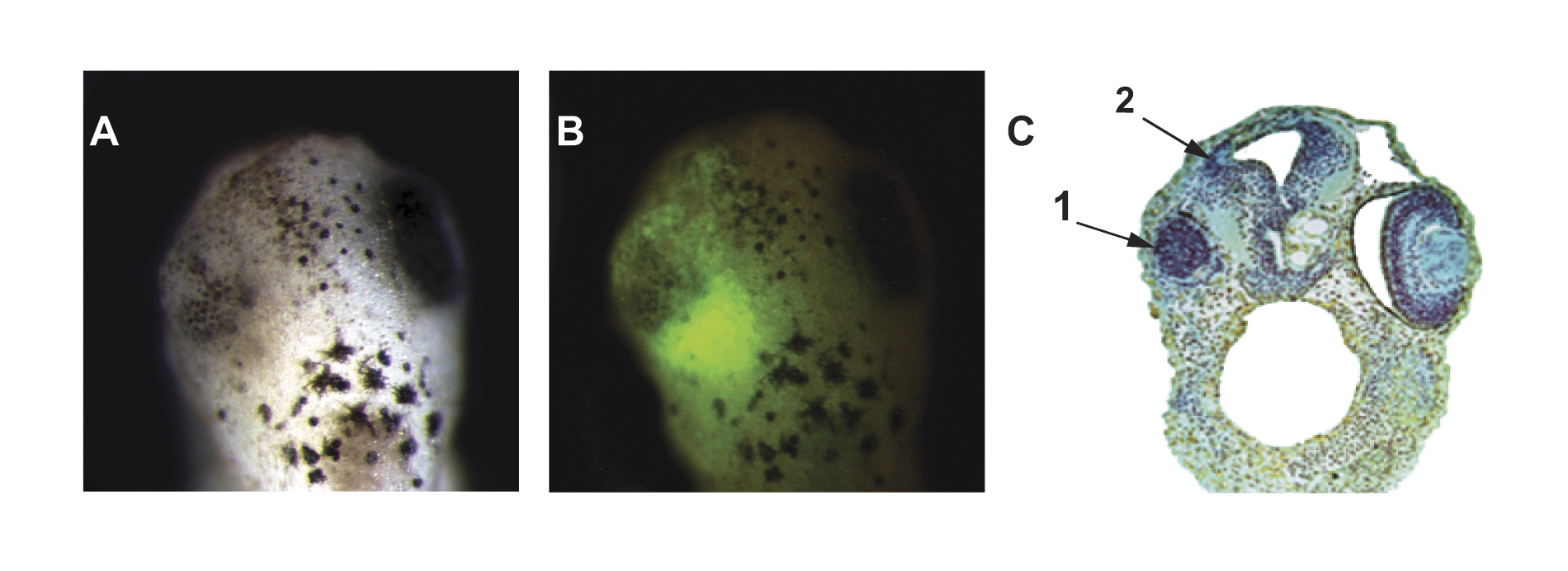

Supplement: Figure S1 — SPC7 antisense morpholino oligonucleotide causes dose-dependent disruption of eye and brain development –30 ng dose. (A) Dorsal view of the head of a stage 35 embryo injected unilaterally with 30 ng MO. The MO injected side, to the left, failed to develop a normal eye. (B) Localization of fluorescein tagged MO in same embryo. (C) Frontal section of embryo shown in (A) and (B) showing a disorganized neural retina and no lens (arrow). The right, uninjected side was unaffected. (TIF) [file pone.0039380.s001.tif]
